# What Role Do Local Grocery Stores Play in Urban Food Environments? A Case Study of Hartford-Connecticut

**DOI:** 10.1371/journal.pone.0094033

**Published:** 2014-04-09

**Authors:** Katie S. Martin, Debarchana Ghosh, Martha Page, Michele Wolff, Kate McMinimee, Mengyao Zhang

**Affiliations:** 1 Department of Nutrition and Public Health, University of Saint Joseph, West Hartford, Connecticut, United States of America; 2 Geography, University of Connecticut, Storrs, Connecticut, United States of America; 3 Hartford Food System, Hartford, Connecticut, United States of America; 4 Department of Allied Health Sciences, University of Connecticut, Storrs, Connecticut, United States of America; University of Washington, United States of America

## Abstract

**Introduction:**

Research on urban food environments emphasizes limited access to healthy food, with fewer large supermarkets and higher food prices. Many residents of Hartford, Connecticut, which is often considered a food desert, buy most of their food from small and medium-sized grocery stores. We examined the food environment in greater Hartford, comparing stores in Hartford to those in the surrounding suburbs, and by store size (small, medium, and large).

**Methods:**

We surveyed all small (over 1,000 ft^2^), medium, and large-sized supermarkets within a 2-mile radius of Hartford (36 total stores). We measured the distance to stores, availability, price and quality of a market basket of 25 items, and rated each store on internal and external appearance. Geographic Information System (GIS) was used for mapping distance to the stores and variation of food availability, quality, and appearance.

**Results:**

Contrary to common literature, no significant differences were found in food availability and price between Hartford and suburban stores. However, produce quality, internal, and external store appearance were significantly lower in Hartford compared to suburban stores (all p<0.05). Medium-sized stores had significantly lower prices than small or large supermarkets (p<0.05). Large stores had better scores for internal (p<0.05), external, and produce quality (p<0.01). Most Hartford residents live within 0.5 to 1 mile distance to a grocery store.

**Discussion:**

Classifying urban areas with few large supermarkets as ‘food deserts’ may overlook the availability of healthy foods and low prices that exist within small and medium-sized groceries common in inner cities. Improving produce quality and store appearance can potentially impact the food purchasing decisions of low-income residents in Hartford.

## Introduction

The availability of nutritious and affordable food can greatly impact chronic disease rates and other critical individual health outcomes in a community [1]–[4]. For example, insufficient access to healthy and affordable food may adversely affect dietary intake and eventually lead to nutrition related negative health outcomes such as obesity, diabetes, and cardiovascular diseases [5], [6]. In urban inner cities, the situation is often thought to be even more critical due to nutritional imbalance caused by easy access to abundant fast-food restaurants on one hand and lack of large supermarket grocery stores on the other hand [7], [8].

Dietary intake is determined by a number of factors, including what types of food items are available (*availability*), at what prices (*affordability*), and proximity to grocery stores (*accessibility*).

Researchers typically describe areas with lack of access to healthy and nutritious food as *food deserts* [4], [9]–[14]. Among the several definitions, the United States Department of Agriculture’s (USDA) description of food desert is the most commonly used, where, census tracts are identified as food deserts if they satisfy the following two conditions of: 1) “*low-income communities*”, based on having a poverty rate of 20% or greater or a median family income at or below 80% of the area median family income; and 2) “*low-access communities*”, based on the determination that at least 500 persons and/or at least 33% of the census tract’s population live more than *one mile* from a supermarket or a large grocery store [15]. There have also been prior attempts to expand the definition and methodology for delineating food deserts for rural areas [16]–[18], urban areas [16], [19]–[23], and by incorporating different measures of accessibility determined by distance, time, and modes of transportation to grocery stores [1], [24]–[26] [20], [27], [28] [29]–[32]. Residents living in food deserts often experience food insecurity, which is defined as limited food access or uncertain ability to acquire acceptable foods in socially acceptable ways [33]. Similar to food deserts, several researchers have also associated food insecurity with a greater prevalence of chronic diseases and health disparities in urban, low-income areas [1], [34]. Recent estimates indicate that approximately 14.5% of all US households experienced food insecurity in 2012 [35].

Few recent studies on food environment, however, have indicated the need to further expand the definition of food deserts by looking more critically at small and medium-sized grocery stores where inner-city residents typically shop for their food items [36], [37]. A primary concern for urban areas, in regards to food accessibility, involves the lack of large-scale retail supermarkets with stocks of nutritious food at affordable prices, compared to predominately white or more affluent suburban neighborhoods [25], [26], [38]–[42]. Due to ‘supermarket redlining’ [43], [44], large chain supermarkets are often disinclined to locate their stores in inner cities and usually pull their existing stores out and relocate them to suburbs. As the stores close, urban residents have to travel further to purchase nutritious and affordable groceries or shop at local medium and small sized grocery stores that may lack healthy food or offer it at higher prices than the larger stores [45], [46]. The situation is often thought to be even more critical for residents with limited access to automobiles or well-connected public transportation. People living in urban communities with restricted access to larger stores pay significantly more (3%–37%) for groceries compared to those living in the suburbs buying the same products at supermarkets [3], [25], [46]–[49]. A study by Raja et al. [37], on the contrary, indicated that small-sized grocery stores sometimes do play an important role in providing healthy, affordable, and culturally appropriate foods in minority neighborhoods.

In addition to cost and availability, ethnically diverse shoppers in low-income areas also value variety and express concerns about the quality of fresh produce in smaller stores [50]. Product variety is often defined as the range of product options available to the consumer, enabling consumer choice [51]–[53]. Even when affordable food sources are present, they have been found to be of poorer quality than those found in wealthy or predominantly white neighborhoods [47], [54]–[58]. The quality of fruits and vegetables is assessed by whether they appear damaged or look unappealing, and often they are less likely to be purchased [52]. The quality of fresh produce and perceived quality of one’s food environment directly affect dietary intake [55], [59]. Therefore a closer inspection of medium to small-sized stores as an alternate source of healthy, good quality, and affordable food items is required in an area where large supermarkets are hard to find.

This study attempts to contribute to the research of nutrition, food access, and urban food systems by including medium and small sized grocery stores in the definition of food deserts and expanding the concept of accessibility to a multidimensional construct [60]. Rather than defining food access as simply physical distance to a large supermarket, here we examine accessibility (distance to stores), availability (variety of food items), affordability (price), quality of fresh produce, and appearance of small, medium, and larger grocery stores in greater Hartford, Connecticut (CT), to provide a more robust and accurate measure of the urban food environment. Defining access to only large supermarkets can overlook the contribution that small to medium-sized groceries can play in an urban food systems [61]. Therefore we also seek to determine how small and medium-sized grocery stores compare with larger supermarkets in regard to the different dimensions of accessibility mentioned above.

### Study Setting: City of Hartford, Connecticut

The City of Hartford in Connecticut (CT), with its diverse demographic, socioeconomic, and health disparity indicators provides a unique opportunity to explore the multidimensional construct of accessibility to not only healthy food but also ‘affordable’ and ‘quality’ food. With a population of 124,893, Hartford has an estimated poverty rate of 32.9%, more than double the US poverty rate of 15% [62], [63]. Almost one-half of children in Hartford live below the poverty line (47.9% for a total of 14,814 children), compared to the US child poverty rate of 21.8%. The unemployment rate in Hartford in April 2013 was 14.8% [64], compared to approximately 7% nationally [65]. The 2011 median household income was estimated at $29,169, which is less than half of the estimated median household income for the county of Hartford, as well below the median for the United States, which was $50,502 [66], [67]. Almost one-third (31.8%) of Hartford households have no vehicle [67].

The youngest members of the Hartford community are at increased risk of diet-related diseases due to nutritionally imbalanced access to foods in their neighborhoods. A 2012 study found that 37% of preschool children in Hartford are overweight or obese, making the prevalence of childhood obesity among preschoolers more than twice as high as Centers for Disease Control and Prevention age and gender body mass index guidelines [68].

The City of Hartford Advisory Commission on Food Policy has historically focused on ensuring that a wide variety of safe and nutritious foods are available for City residents. The Advisory Commission, which was formed in 1991, was one of the first commissions in the US to make nutritious and affordable food a priority. One of the four goals of the Commission is “To ensure that the price of food in the city remains reasonably close to the average price existing in the balance of the state” (Bylaws section 2–327 part b, goal 3 Hartford, CT, 1991) [69]. The Commission has conducted food price surveys in grocery stores on an ad-hoc basis since 1996 and has endorsed this research as supportive of Commission goals. Two of this study’s researchers currently serve as Commissioners. The ultimate goal of this empirical research is to inform policies related to healthy food access and food insecurity in urban communities such as Hartford.

## Study Goals, Objectives, and Research Questions

The objectives of this empirical study were to examine the *accessibility* (proximity to grocery stores), *affordability* (food prices) and *quality* (whether fruits and vegetables appear damaged or look unappealing and overall appearance of the store) of healthy food sold at large, medium, and small-sized grocery stores in Hartford and within a two-mile radius of the city. The specific research questions are:

1. Do Hartford residents have access to large, medium, and small sized grocery stores?
2. Do availability, price and quality differ by urban/suburban location?
3. Do availability, price and quality differ by store size?

The overall goal was to assess whether low-income households in Hartford who do not own or have limited access to a car would be able to access stores with quality and affordable food. The hypothesis was that small and medium-sized stores that are more common in urban areas could be a potential alternative to scarce presence of large supermarkets. Results can then help inform policy recommendations by the Advisory Commission on Food Policy to City leaders regarding where city resources should be allocated to increase access to healthy foods.

## Methodology

**Figure 1:**
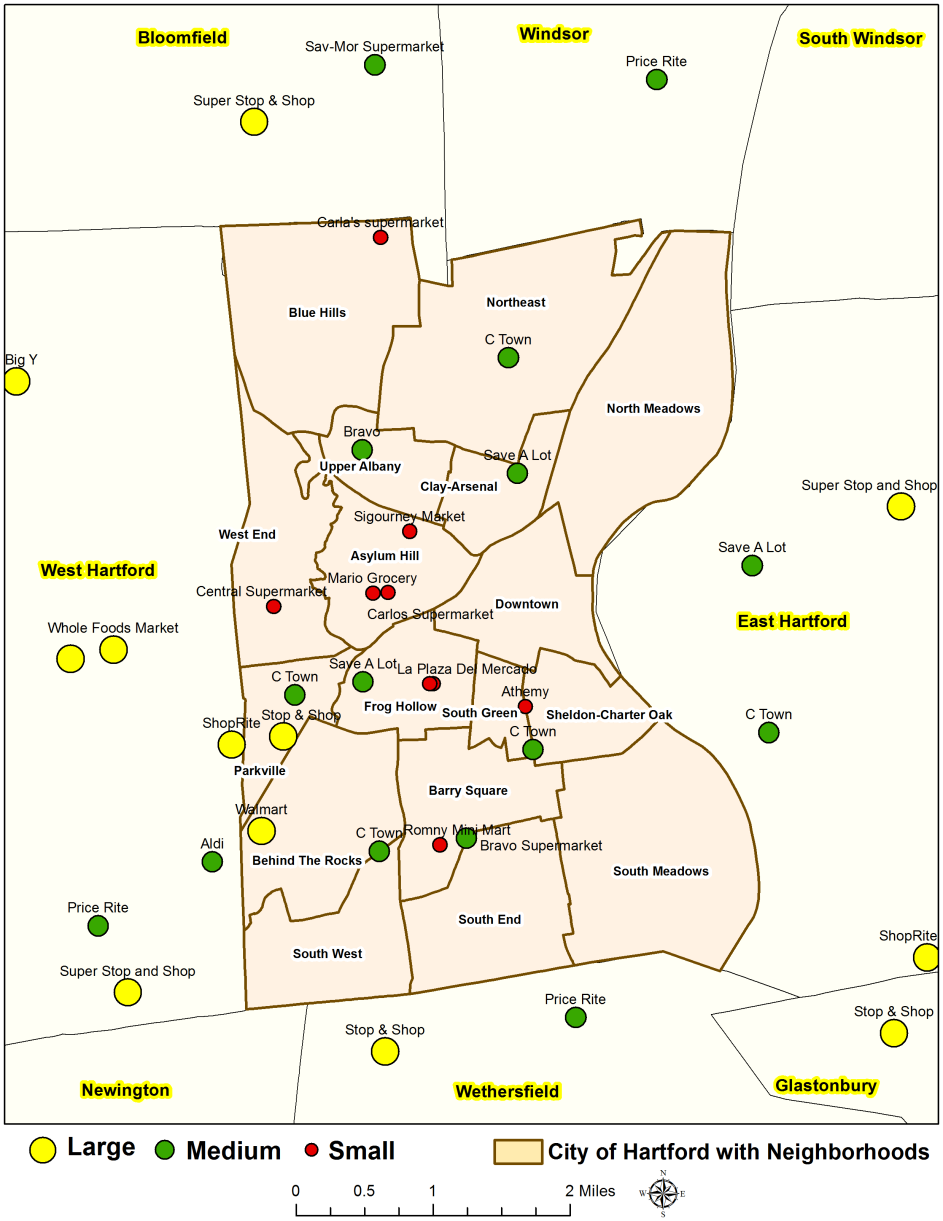
Location of surveyed stores in the City of Hartford, Connecticut.

Between October 2012 and March 2013, surveys were conducted in 36 stores in Hartford and within a 2-mile radius of Hartford including parts of several adjacent suburban towns (See Figure 1). The 2-mile radius was drawn from the outline of the City of Hartford. We used a two-mile radius for two reasons: first, the residents of Hartford often shop outside the city limits, and second, to minimize errors from edge effects in the subsequent mapping and spatial analysis. This represents an inventory of all small, medium, and large groceries. The details of selection criteria, size, characteristics, and example of each type of stores are described later. We did not seek formal (or written) permission to conduct these surveys for the following reasons: a) the selected grocery stores are open to the public and do not belong to any private land or property; b) the researchers followed a participant observation data collection technique where they only observed and took notes and did not interact with any human subjects (other shoppers or store workers) during the survey; c) as detailed later, the survey instrument required taking notes of the sale prices and the quality of various food items, which are public information (Refer to Price Survey Instrument); and d) all the researchers have training in conducting ethical research. This survey did not involve endangered or protected species.

Stores were identified based on previous research on the Hartford food environment [61], [70] and using a variety of methods to ensure sample completeness, including online yellow pages, business listings, and more importantly “ground-truthing” of driving through neighborhoods to identify stores. This represents an inventory of all medium, and large size grocery stores in the study area, as well as several smaller stores that, based on inventory and selection, operate as grocery stores rather than convenience stores. The City of Hartford, like many other urban settings, has a number of small and medium-sized grocery stores that stock a full array of groceries and are larger than typical corner stores, but smaller than supermarkets.

These “small” stores are defined as independent food stores between 1,000 and 2,500 ft^2^ based on field measurements from previous research in Hartford [70]. Medium-sized grocery stores are approximately 15,000–39,999 ft^2^ and generally stock a limited number of custom-brand high-volume food items at discount [71]. Examples of stores in this category include Save-A-Lot and Bravo. Large supermarkets range from 40,000 to 80,000 ft^2^ and typically include delicatessens, bakeries, pharmacies, and general merchandise in addition to groceries. Examples of stores in this category include ShopRite, Big Y, and Stop and Shop. Stores were also categorized based on whether they were certified to accept vouchers for the Special Supplemental Nutrition Program for Women, Infants and Children (known as WIC) based on data from the Connecticut Department of Public Health. WIC certified stores are required to stock a limited number of fruits and vegetables so they may be more inclined to have produce available. Two of the grocery stores that fit the study criteria were no longer in business and were removed from the sample. Convenience stores, such as bodegas and corner stores, were not included in the study group. Trained researchers participated in a practice survey at several grocery stores prior to the start of data collection. Researchers subsequently completed the data collection in all stores in the study group.

### Physical Accessibility and Mapping

To address research question #1 (access to large, medium, and small size grocery stores in Hartford), we used Geographic Information System (GIS) for mapping and spatial analysis. To begin, the 36 surveyed stores were geocoded. Geocoding is the process by which a postal address or any other locational information is associated with geographic units such as points (latitude and longitude coordinates), postal zip codes, or US Census block groups or census tracts [72]. For this study the postal addresses of the surveyed stores were geocoded to points (latitude and longitude).

To measure accessibility (or distance to stores), we used ancillary GIS data sets downloaded from various sources. These data sets are: a) Boundaries of US Census block groups [73], b) Boundaries of neighborhoods in Hartford [74], c) Detailed road network data [73], and d) Location of bus Stops [74]. The transit data was limited and had only locations of bus stops with no additional information of time schedule, routes, or transfers.

Next, we calculated distances on road network from the population centroid of a block group in Hartford to the nearest store using the ‘closest facility’ function of the Network Analyst Extension in ArcGIS 10.1 [75]. This function measures the distance (sometimes shortest route) of traveling between incidents (population centroid of a block group in our study) and facilities (surveyed store) and determines which are nearest to one another [75]. This measurement is different than calculating Euclidean distances or “as the crow flies” distance between two points [76] and is a better reflection of how people travel and perceive distance. Two Hartford neighborhoods (North and South Meadows) that are non-residential were excluded from the analysis.

Once the distances were calculated from each of the centroids, we then created a continuous distance surface by Inverse Distance Weighting interpolation technique to calculate distances to the nearest stores [72]. Using a spider-diagram function we calculated how many stores have at least one bus stop within a distance of 0.25 miles [77]. This distance cut-off was determined based on the inconvenience of walking over a quarter mile with groceries. Spider Diagram function creates a line representing the shortest distance between centers (surveyed stores in our study area) and the closest destinations (locations of bus stops in our study area). Finally, the variability of prices and quality of available food by store size (large, medium, and small) and location (Hartford versus suburban) were also mapped for better visualization and understanding.

### Survey Instrument

To address the above-mentioned research questions #2 (do availability, price and quality differ by store size?) and #3 (do availability, price and quality differ by urban/suburban location) data were collected using a grocery store survey of a market basket of food. The survey instrument is based on previous price surveys conducted by the Hartford Advisory Commission on Food Policy [78]. (Refer to File S1 - Price Survey Instrument). The instrument included a list of 25 grocery items, categorized by milk, cheese, fresh produce, protein, bread, canned and staple items, and beverages. Items were chosen to represent a standard basket of healthy food items that could be used to prepare breakfast, lunch, and dinner and included produce items that would be most likely available during all seasons. The standard size of each item was listed on the survey. The least expensive brand or option available in the store was recorded as an item’s price; sale price was not used unless it was the only price posted, and if so, the sale was noted in the comments field. Produce price was calculated by an item’s price per weight and standard measures for each item were noted on the survey instrument. If an item was not present in the store at the time of the survey, researchers noted it as unavailable.

Quality of fresh produce was measured on a 1 to 4 Likert scale. A score of 1 indicated most of the item was of poor quality, such as brown, bruised, overripe or wilted. A score of 2 indicated more poor than good; 3 indicated more good than poor. A score of 4 meant most of the item was of good quality, very fresh, no soft spots, and of excellent color. Each store was also rated on internal and external appearance, and was scored from 1–4 as poor, fair, acceptable, or good respectively. Internal quality included the overall appearance, lighting, cleanliness, and organization inside the store. External quality was based on the appearance, lighting, perceived safety, and parking of the store’s exterior. Researchers assessed several stores as a team and discussed the scoring as a group to ensure standardization for measuring and evaluating store appearance and food quality.

### Data Analysis

Data were analyzed using PASW v.18. Some variables were not normally distributed including internal and external appearance, produce quality, and availability of food items. For these variables, non-parametric tests were used. Price was normally distributed and continuous, so t-tests and ANOVA were run to compare means. Significance was determined at p<0.05. Comparisons between Hartford and suburban stores were made using Fisher’s exact chi-square test for small cell sizes and Mann-Whitney U test for non-parametric data. Quality measures were included as continuous variables and also dichotomized for ease of interpretation. Scores for internal and external appearance were dichotomized between low (1–3) and high (4) scores, and produce quality between low (0–35) and high scores (36–40) based on the distribution of the data. When comparing stores by the three store sizes, comparisons were made using Kruskal-Wallis test for independent samples for non-parametric data.

## Results

### Distribution of Grocery Stores in Greater Hartford

The sample includes 36 large, medium, and small-sized grocery stores, including 20 stores (55%) within the city limit of Hartford and 16 stores (45%) in the adjacent suburban towns, located in the 2-mile radius from the city boundary. The majority of stores (64%) are certified to accept vouchers for WIC. No significant differences exist in the availability of WIC certified stores, neither between the City of Hartford and the suburban stores, nor by store size.

### Accessibility to Grocery Stores in Hartford

**Figure 2:**
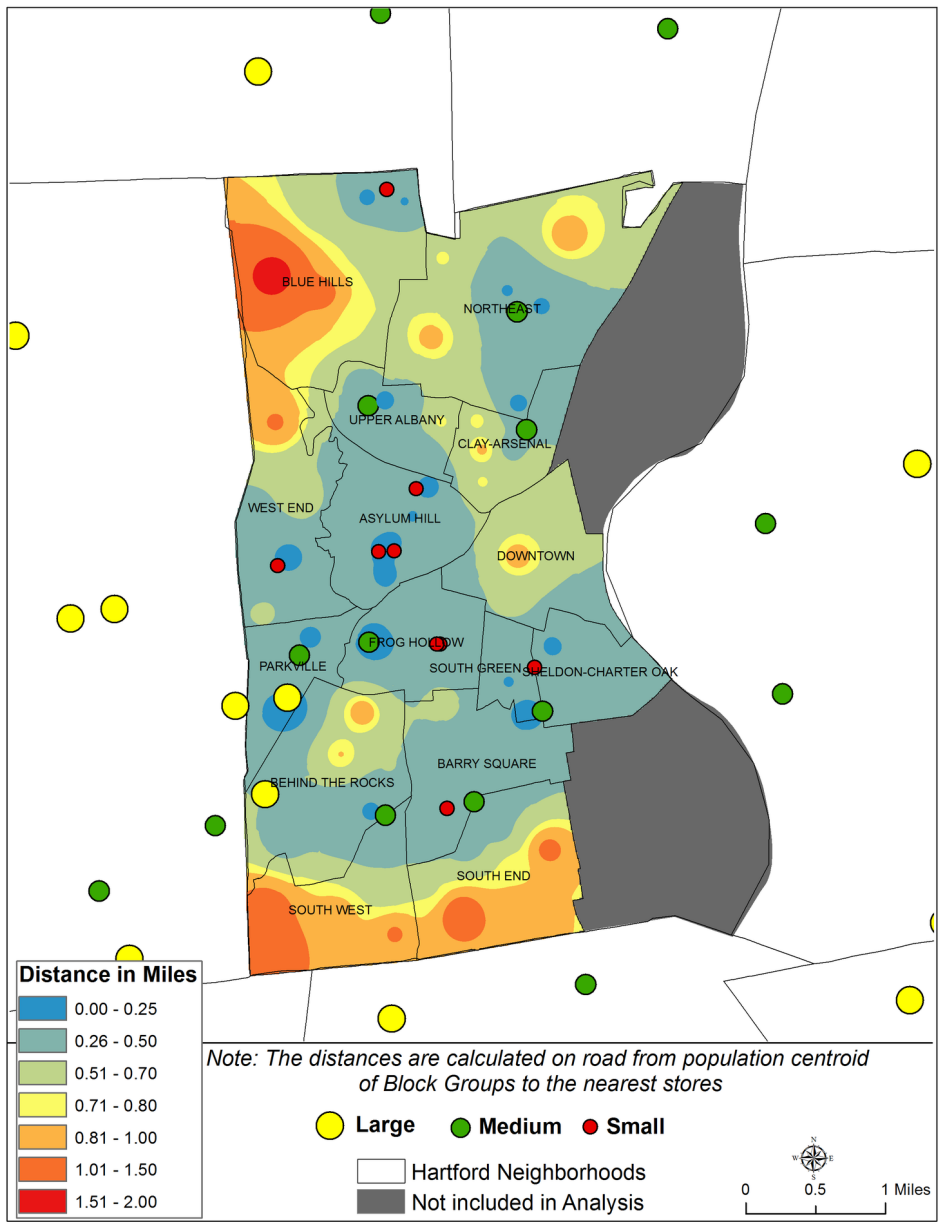
Distance (in miles) to stores from the population centroids of US Census Block Groups.

Distance in miles from the population centroid of the census block groups to the nearest large, medium, and small sized grocery stores in the study area is shown in Figure 2. Based on the location of the stores, residents living in the central and south central part of the City of Hartford are on average 0.25 to 0.50 miles distance away from stores. Within this area there are a few pockets of lesser accessibility, where residents have to travel more than 0.5 mile to a grocery store. Residents living in Downtown Hartford are at a distance of 0.75 to 1 mile from a store indicating a situation of urban ‘food desert’. Moving farther away from the south and central areas of the City, spatial accessibility to any grocery stores decreases. In some areas, especially in the neighborhoods of Blue Hills in the North, and South West and South End in the South there is not a single grocery store within a distance of 2 miles. Out of 93 block groups in the City of Hartford, 44% are at an average distance of 0.25 to 0.50 miles from a store (Table 1). The maximum distance from the center of a block group to the nearest surveyed store is 1.98 miles. The minimum is 0.05 miles and the average distance is 0.57 miles.

**Table 1:** Distance (in miles) from the center of the US census block groups to the nearest grocery store.

Distance Groups (in miles)	Number of Block Groups (N = 93) (%)
0.00–0.25	17 (18.3)
0.26–0.50	41 (44.1)
0.51–0.70	13 (14.0)
0.71–0.80	6 (6.5)
0.81–1.00	8 (8.6)
1.01–1.50	7 (7.5)
1.51–2.00	1 (1.1)

We further explored the accessibility to grocery stores in terms of public transportation in the City of Hartford, using the location of bus stops. All 36 stores have at least one bus stop (very often more than one) within a distance of 0.25 miles from the location of the stores. This is true for all the store sizes (large, medium, and small) and locations (Hartford versus Suburban). Four stores have no bus stop within a distance cut-off of 0.1 miles, all of which are large sized grocery stores. This could be due to larger parking lots for large-sized grocery stores, which restricted the locations of bus stops beyond 0.1 miles.

### Internal and External Appearance/Quality

Significant differences exist between stores in Hartford compared to the suburbs regarding quality of fruits and vegetables, and the internal and external appearance of the stores. Hartford stores were significantly more likely to have low internal store quality, a measure of appearance, lighting, cleanliness and organization. Only six stores (32%) in Hartford had high internal quality scores compared to 12 suburban stores (71%, p = 0.02). Of the six stores in Hartford with high internal quality, one was large, two were medium, and three were small sized stores (Table 2).

**Table 2:** Characteristics of Stores: Comparison between Hartford and Suburban location.

Characteristic	Subcategory	Hartford	Suburban
		Stores	Stores
		Number (%)	Number (%)
**Total Number of Stores**		19 (52.8)	17 (47.2)
**Store Size**			
	Small	9 (47.4)	1 (5.9)
	Medium	8 (42.1)	7 (41.2)
	Large	2 (10.5)	9 (52.9)**
**WIC Certified**		13 (68.4)	10 (58.8)
**Quality and Appearance**			
	High internal quality score of 4	6 (31.6)	12 (70.6)*
	High external quality score of 4	2 (10.5)	11 (64.7)**
	High Produce quality scores from 36–40	8 (42.1)	14 (82.4)*
		**Average (SD)**	**Average (SD)**
	Internal quality (scale of 1–4)	0.1 (0.8)	3.7 (0.5) *
	External quality (scale of 1–4)	2.5 (0.7)	3.6 (0.6) **
	Produce quality (total score of 40)	34.9 (3.8)	37.9 (3.5) *
**Availability of 25 food items**		22.8 (3.6)	24.7 (0.6)
**Total Price of 25 food items**		$47.77 (5.33)	$48.46 (10.5)

SD = Standard Deviation.

* = 0.01<p<0.05, ** = *P*<0.01.

**Figure 3:**
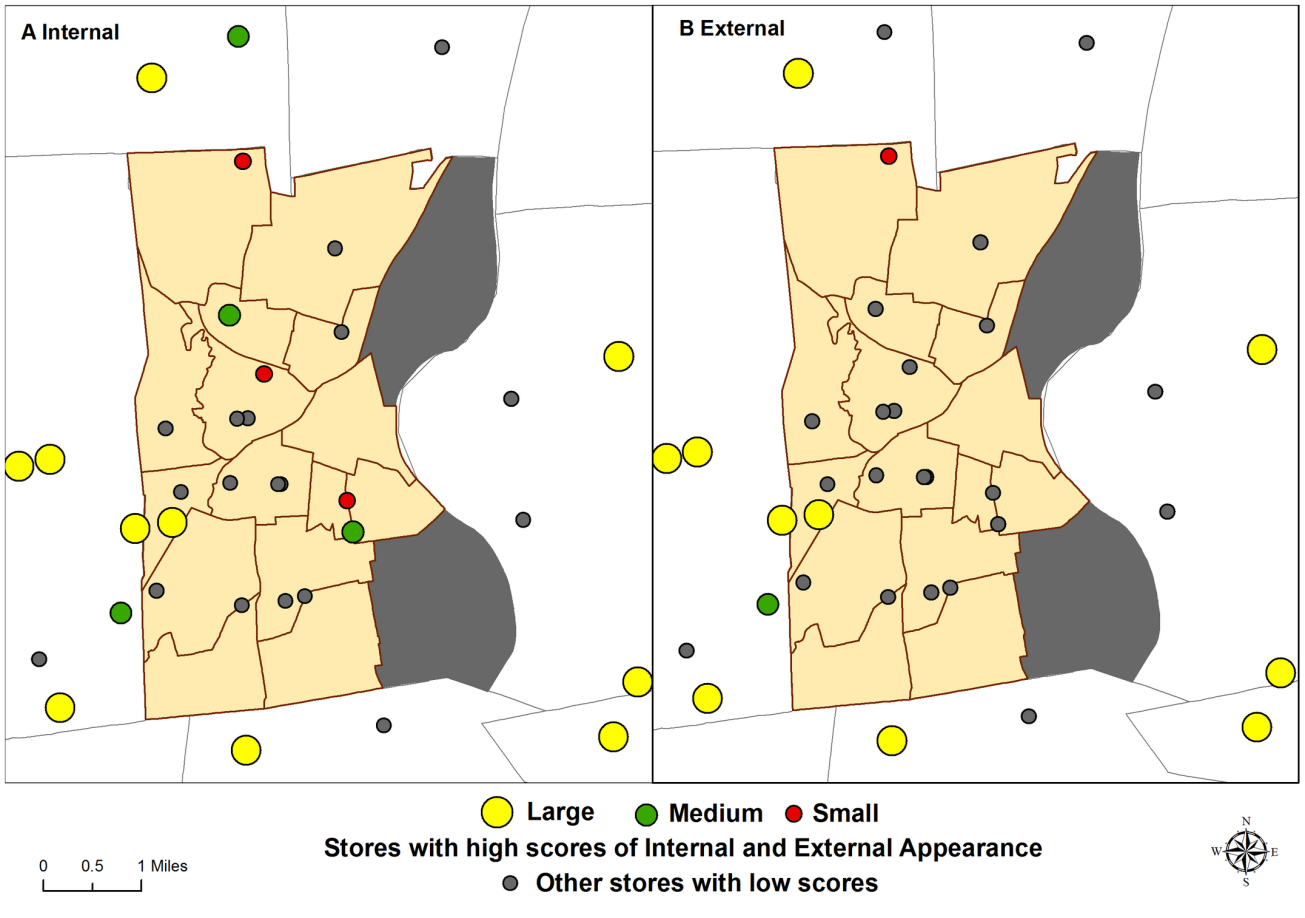
Stores with high scores (Score = 4) of Internal and External appearance.

Similarly, Hartford stores scored much lower on external store quality, a measure of lighting, perceived safety and parking outside the store. Hartford stores were significantly less likely to have high external appearance scores, with only two Hartford stores (11%) receiving high scores, compared to 11 suburban stores (65%, p<0.01). Only one small sized grocery store located in the northern neighborhood of Hartford had high scores for both internal and external quality or appearance. This is notable because this area has a high proportion of low-income minority population with limited access to large supermarkets. Figure 3 displays the variation of internal and external appearance scores by location (Hartford versus suburban) and size of stores (large, medium, small). A clear spatial pattern exists where stores, irrespective of their size, had higher scores for both internal and external appearance in the suburban towns.

### Produce Quality

**Figure 4:**
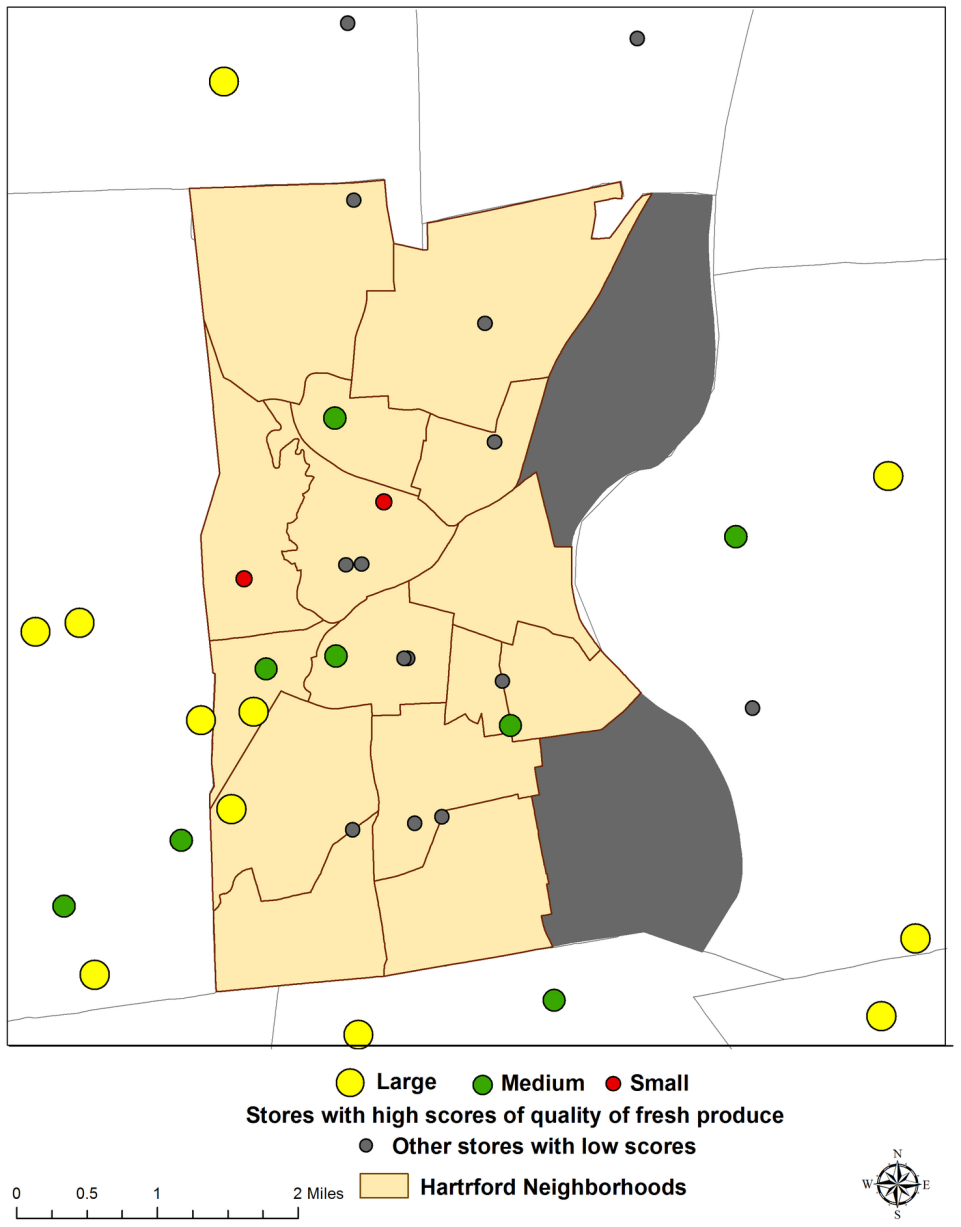
Stores with high scores (Score = 4) of produce quality.

Quality of fresh fruits and vegetables also differed significantly between Hartford and suburban stores. Eight Hartford stores (42%) received high produce quality scores, compared with 14 suburban stores (82%, p = 0.02), (Figure 4). In the City, two small grocery stores had high quality produce. Three medium suburban grocery stores had low scores for produce quality, which is contrary to current literature and assumptions that suburban neighborhoods will carry high quality healthy food (Table 2).

### Price and Availability of Food Items

No significant differences were found for the price and availability of food items between stores in Hartford and surrounding suburbs. The average price of the 25 items for the overall sample was $48.10; with an average price of $47.77 in Hartford stores, and $48.46 in the suburbs. The majority of stores (64%) carried all items, 22% were missing 1–2 items, and 14% had fewer items. A noticeable exception was two small stores in Hartford that only carried 14 of the 25 items.

### Comparisons by Store Size

Significant differences existed for quality, availability, and price, between small to medium, medium to large and between small and large stores (Table 3). Large stores had better scores for internal, external and produce quality variables. Medium-sized stores had greater availability compared to small stores, and had lower prices than either small or large stores.

**Table 3:** Characteristics of Stores: Comparison Between Small, Medium and Large sized stores.

Characteristic	Small Stores	Medium Stores	Large Stores
	Number (%)	Number (%)	Number (%)
**Total number of stores**	10 (27.8)	15(41.7)	11 (30.6)
**WIC Certified**	4 (40)	11 (73.3)	8 (72.7)
	**Average (SD)**	**Average (SD)**	**Average (SD)**
**Internal quality (scale of 1–4)**	3 (1.1)	3.3 (0.5)* ^b^	3.9 (0.3)* ^c^
**External quality (scale of 1–4)**	2.6 (0.8)	2.7 (0.6)** ^b^	3.8 (0.6)** ^c^
**Produce quality (total score of 40)**	33.4 (3.7)	35.7 (3.7)* ^b^	39.7 (0.5)** ^c^
**Availability of 25 food items**	21.7 (4.3)	24.5 (0.9)* ^a^	24.5 (1.8)
**Total Price of 25 food items**	52.47 (5.0)	41.94 (5.5)** ^ab^	52.52 (8.1)

SD = Standard Deviation.

* = 0.01<p<0.05, ** = *P*<0.01.

a = significant difference between small and medium stores.

b = significant difference between medium and large stores.

c = significant differences between small and large stores.

## Limitations

The analysis of transit accessibility to stores using just the location of bus stops has limitations and does not capture the complexity of transit network. We only considered the number of bus stops within a distance of 0.25 miles from a store and did not have other supporting information such as number of buses, routes, frequency, schedules, or transfers. This additional information would provide a better measure of accessibility of the grocery stores by public transport.

The scale used to assess internal, external, and produce quality may not have captured the full range of variation among items or store quality. The scale was a subjective measure of quality, although clear guidance on how to rate store quality and fresh produce was given to the survey team. Data was collected from stores in one urban area as a case study; therefore results may not be generalizable to other communities. Due to the small sample, it is possible that some insignificant findings may be significant with a larger number of stores. For example, a larger percentage of medium-sized stores were WIC certified compared to small stores, yet this was not statistically significant. Surveys were administered at one point in time. We assume that quality, availability, and price are fairly stable over time, although produce supply may vary seasonally.

## Discussion and Conclusion

Conventional literature and groundwork on urban food systems emphasizes limited access to healthy food in inner-city low-income neighborhoods compared to surrounding suburbs, with fewer large supermarkets and higher food prices [42], [79]. In contrast, results from this study, based in Hartford, Connecticut, found that healthy foods are equally available and sometimes less expensive in local stores in the City compared to suburban stores. Although physical accessibility to grocery stores is not uniform in all Hartford neighborhoods, most Hartford residents live within a 0.5 to 1 mile distance to a grocery store. However, there are a few pockets that would be considered *food deserts* – with no grocery store within a 1-mile or even 2 miles radius.

One of the major findings is the significant variability of quality of produce and store appearance, with Hartford stores faring much worse than the suburban stores. The lower quality may impact customers’ willingness to shop in these stores or to purchase fruits and vegetables that contribute to a healthy diet, which can help prevent or mitigate chronic diseases [50]. Improving food quality and appearance of smaller stores in urban inner cities can potentially impact the food purchasing decisions of low-income residents in Hartford and a potential improvement to food insecurity [59].

Focusing solely on large supermarkets may underestimate food access in urban, low-income cities where medium and small-sized grocery stores are more prevalent [26]. Classifying areas with few large supermarkets as food deserts may overlook the availability of healthy foods that exist within small and medium-sized groceries found in inner cities [61]. Results from this study document the availability and lower prices found in the City of Hartford and shed light on the important role that medium-sized grocery stores play in the accessibility, availability and affordability of healthy food in urban areas.

Many of the store owners from small and medium-sized markets in Hartford live locally. Therefore, efforts to improve the business infrastructure and sales of these markets will help support the local economy, which is in line with the principles of healthy, sustainable food systems. In comparison, large supermarkets tend to be owned by national or often international companies where revenues are not reinvested into the city. Studies have shown that store owners’ personality and established friendships between owners and patrons fosters store loyalty, especially in neighborhoods without a large supermarket [80], [81]. City leadership may want to consider investing in façade improvements, such as internal and external store appearance, and ways to improve produce quality to boost the local economy and potentially improve food purchasing and consumption patterns of low-income residents. These improvements would make the role of small to medium-sized local grocery stores in the urban food system critical in addressing the issues of food deserts in the underdeveloped parts of the city.

Polices and logistics surrounding the opening of large chain supermarkets in impoverished neighborhoods are not easy to implement partly due to a lack of a stable markets and partly due to lack of infrastructure related to easy access to highways, large loading docks for large trucks to unload, and distribution networks. Recent research also indicates that simply placing a large supermarket in a low-income urban community did not improve dietary intake [84]. Large stores are often disinclined to locate their stores in inner cities and usually pull their existing stores out and relocate them to suburbs [43], [44]. As the stores close, impacted urban residents have to travel further to purchase nutritious and affordable groceries or turn to small and medium-sized local grocers. In the City of Hartford, eleven of thirteen chain supermarket stores (almost 85% of the stores) have left the city between 1968 and 1984 [82]. Recently, Russell and Heidkamp [83] found that a food desert was created when Shaw’s (http://www.shaws.com) was closed in the city of New Haven, CT, which has similar socioeconomic, demographic, and health disparity characteristics to that of Hartford.

This research contributes to nutrition, access to healthy food, and sustainable food systems literature by emphasizing differences based on location and store size. Results from this study can help influence recommendations for the Hartford Advisory Commission on Food Policy and promote action from other city agencies to improve food quality and store appearance in Hartford stores. In addition, this research may contribute to decisions by policymakers in other urban communities to allocate resources to improve existing small and medium sized markets before creating new large stores. Improving the quality of food, and store appearance in urban stores are relatively more plausible than locating a large chain supermarket. These improvements may influence purchasing decisions of low-income households who lack adequate transportation, and improve poor dietary intake linked to health disparities and food insecurity.

## Supporting Information

File S1 **Price Survey Instrument.**
(DOC)
