# Supplementary material for: What Role Do Local Grocery Stores Play in Urban Food Environments? A Case Study of Hartford-Connecticut
Source: PLoS One. 2014 Apr 9;9(4):e94033. doi: 10.1371/journal.pone.0094033 (PMC3981752; doi:10.1371/journal.pone.0094033)
Supplement: File S1 — Price Survey Instrument. (DOC) [file pone.0094033.s001.doc]

**Grocery Store Price Survey**

**Instructions**

- Capture the price for the least expensive brand/option available.
- Include brand name in comments field.
- If the indicated size is not available, note the size used in the comments field.
- If the indicated item is not available, note that in the comments field.

**Pricing**

1. If price is not available, ask an employee at the cash register or at customer service. Wait until all of the measures have been completed before asking the price of the items that are needed. There may be exceptions to this (i.e., you are in the produce section and there is no price shown but an employee is working there), so use your judgment.

2. Do not use a sale price unless it is the only price posted and write “sale price” in comments.

**General Completion Tips**

Remember to follow the tips below to decrease the data cleaning time later.

1. Write legibly.

2. Check your work. Make sure you have complete information.

**Notes: please write any additional notes below to describe the availability, price or quality of the food, or impressions of the store appearance, either in the store or externally.**

______________________ _______________________ _______________________ _____________

**Store Name** **Address** **Reviewer Name** **Date**

| **Dairy** | | | | |
| --- | --- | --- | --- | --- |
| ***Type*** | ***Size*** | ***Price*** | ***Comments*** | |
| **Milk** |  |  |  | |
| Skim or 1% | Half- Gallon |  |  | |
| 2% | Half- Gallon |  |  | |
| **Cheese** | | | | |
| American cheese slices | 12oz |  |  | |
| **Fresh Produce** | | | | |
| **Quality: Write the number that best describes the overall quality of the produce for each item.**  1 = Poor: all or most of the item is of poor quality (brown, bruised, overripe, wilted); 2 = Mixed quality; more poor than good;  3 = Mixed quality; more good than poor; 4 = good: All or most of the item is of good quality (very fresh, no soft spots, excellent color) | | | | |
| **Fruit** | ***Size*** | ***Price*** | ***Quality, 1- 4*** | ***Comments*** |
| Apples | Per lb |  |  |  |
| Bananas | Per lb |  |  |  |
| Oranges | Per lb |  |  |  |
| Grapes | Per lb |  |  |  |
| **Vegetables** | | | | |
| Carrots | Per lb bag |  |  |  |
| Tomatoes | Per lb |  |  |  |
| Red Peppers | Per lb |  |  |  |
| Broccoli | Per lb |  |  |  |
| Iceberg Lettuce | Per head |  |  |  |
| Celery | Per bunch |  |  |  |
| **Protein** | | | | |
| Ground Beef, 80% lean | Per lb (<2 lbs) |  |  | |
| Chunk Light Tuna in Water | 5 oz can |  |  | |
| Eggs, large | 1 dozen |  |  | |
| **Bread** | | | | |
| 100% Whole Wheat Bread | 1 loaf |  |  | |
| **Canned and Staple Items** | | | | |
| Rice – White, medium | 2 lbs |  |  | |
| Canned beans, pinto, navy, kidney or cannellini | 15 oz (Goya) |  |  | |
| Canned tomatoes | 15 oz |  |  | |
| Spaghetti Sauce | 25 oz |  |  | |
| Pasta, Spaghetti | 1 lb |  |  | |
| Peanut Butter | 18 oz |  |  | |
| **Beverages** | | | | |
| 100% Orange Juice | Half-gallon |  |  | |
| 100% Apple Juice | Half-gallon |  |  | |

**Internal Quality: Appearance, lighting, cleanliness, organization 1=poor 2=fair 3=acceptable 4 =good**

**External Quality: Appearance, lighting, safety, parking 1=poor 2=fair 3=acceptable 4 =good**
